# Trait‐abundance relation in response to nutrient addition in a Tibetan alpine meadow: The importance of species trade‐off in resource conservation and acquisition

**DOI:** 10.1002/ece3.3439

**Published:** 2017-11-03

**Authors:** Huiying Liu, Ying Li, Fei Ren, Li Lin, Wenyan Zhu, Jin‐Sheng He, Kechang Niu

**Affiliations:** ^1^ Department of Ecology College of Urban and Environmental Sciences Key Laboratory for Earth Surface Processes of the Ministry of Education Peking University Beijing China; ^2^ Key Laboratory of Adaptation and Evolution of Plateau Biota Northwest Institute of Plateau Biology Chinese Academy of Sciences Xining Qinghai China; ^3^ University of Chinese Academy of Sciences Beijing China; ^4^ Department of Biology Nanjing University Nanjing China

**Keywords:** community assembly, functional trait, leaf economic spectrum, leaf phosphorus concentration, species relative abundance

## Abstract

In competition‐dominated communities, traits promoting resource conservation and competitive ability are expected to have an important influence on species relative abundance (SRA). Yet, few studies have tested the trait‐abundance relations in the line of species trade‐off in resource conservation versus acquisition, indicating by multiple traits coordination. We measured SRA and key functional traits involving leaf economic spectrum (SLA, specific leaf area; LDMC, leaf dry matter content; LCC, leaf carbon concentration; LNC, leaf nitrogen concentration; LPC, leaf phosphorus concentration; Hs, mature height) for ten common species in all plots subjected to addition of nitrogen fertilizer (N), phosphorus fertilizer (P), or both of them (NP) in a Tibetan alpine meadow. We test whether SRA is positively related with traits promoting plant resource conservation, while negatively correlated with traits promoting plant growth and resource acquisition. We found that species were primarily differentiated along a trade‐off axis involving traits promoting nutrient acquisition and fast growth (e.g., LPC and SLA) versus traits promoting resource conservation and competition ability (e.g., large LDMC). We further found that SRA was positively correlated with plant height, LDMC, and LCC, but negatively associated with SLA and leaf nutrient concentration irrespective of fertilization. A stronger positive height‐SRA was found in NP‐fertilized plots than in other plots, while negative correlations between SRA and SLA and LPC were found in N or P fertilized plots. The results indicate that species trade‐off in nutrient acquisition and resource conservation was a key driver of SRA in competition‐dominated communities following fertilization, with the linkage between SRA and traits depending on plant competition for specific soil nutrient and/or light availability. The results highlight the importance of competitive exclusion in plant community assembly following fertilization and suggest that abundant species in local communities become dominated at expense of growth while infrequent species hold an advantage in fast growth and dispersals to neighbor meta‐communities.

## 1 INTRODUCTION

One of the central goals in ecology is to understand why some species are common and others are rare in a particular habitat (Preston, 1948). From a perspective of niche‐based community assembly, abundant species are those with the best life history strategy in being well‐adapted to the local environmental conditions (Bazzaz, 1996; Grime, 2006; Tilman, 1988). As species life history strategy could be quantified by multiple trait dimensions (Reich, 2014; Violle et al., 2007; Westoby, Falster, Moles, Vesk, & Wright, 2002; Wright, Reich, Westoby, Ackerly, & Baruch, 2004), plant functional traits are expected to determine species relative abundance (SRA; Grime, 2006; Shipley, 2010; Tilman, 1988). Indeed, several field experiments have successfully linked functional traits to SRA in local communities by taking account of dispersal effect (Cornwell & Ackerly, 2010), demographic stochasticity (Shipley, Vile, & Garnier, 2006) and environmental dependence of trait combination (Cornwell & Ackerly, 2010; Yan, Yang, Chang, & Wang, 2013). However, until now how environmental changes affect the relationships between traits and abundance remains uncertain, especially in grassland subject to fertilization and grazing. In addition, most studies have investigated the relations between single trait and SRA, and few studies have paid attentions to the covariations among multiple traits reflecting species trade‐offs in life history strategy in a changing environment (Díaz et al., 2016; Laughlin, 2014; Reich, 2014). The relations between multiple traits and SRA may be very different from the signal trait‐abundance relation because they mirrored more aspects of species performance under changing conditions (Marks & Lechowicz, 2006; Reich, 2014).

In competition‐dominated communities, abundant species are those with a better competitive ability for limited resources (Bazzaz, 1996; Grime, 2006; Tilman, 1988). In contemporary theory to explain the species coexistence and community assembly, SRA in local is driven by the balance between stabilizing niche differences and fitness differences (Chesson, 2000; Hille Ris Lambers, Adler, Harpole, Levine, & Mayfield, 2012) that can be tied to variations in functional traits (Kraft, Godoy, & Levine, 2015). Previous studies have shown that nutrient addition decrease species diversity through competitive exclusion (Borer et al., 2014; Demalach, Zaady, & Kadmon, 2017). In the competition‐dominated communities, traits promote fitness differences, which are associated to SRA. Specifically, traits promoting resource conservation and competitive ability are expected to have an important influence on SRA. For instance, tall plants, and/or with large leaf biomass allocation, usually take advantage in competing limited light resource and would be abundant in fertilized communities (Harpole & Tilman, 2006; Hautier, Niklaus, & Hector, 2009; Liu et al., 2013; Niu, Luo, Choler, & Du, 2008). Accordingly, we expected abundant species do better when they use fixed N efficiently in N‐limited community when P fertilizer was added, thus resulting in a significant relationship between SRA and traits that promote conserve use of fixed N, for example, low leaf nutrient and high LDMC (Reich, 2014).

Additionally, any particular trait in turn is constrained by its functional linkages with other traits. When taking into account covariation among multiple traits, the relationship between SRA and individual trait could be weakened or even altered (Marks & Lechowicz, 2006; Palmer, Stanton, & Young, 2003; Reich, 2014). A key trade‐off involved in plant competition is that high competitive ability and resource conservation often at the expense of resource acquisition for growth (Bazzaz, 1996; Grime, 2006; Tilman, 1988). It is specifically indicated by coordination among multiple traits involved in leaf economics spectrum, that is, species primarily differentiated along a trade‐off axis, which corresponds to traits promoting rapid growth (e.g., high leaf nutrient and SLA) versus those promoting long leaf life (e.g., large leaf dry matter content, LDMC) for resource conservation (He et al., 2009; Reich, 2014; Wright et al., 2004).

In short, we hypothesize that SRA should be positively associated with traits promoting resource conservation and competitive ability (e.g., plant height and LDMC), but negatively related to traits promoting resource acquisition and fast growth (e.g., high leaf nutrient and SLA) in competition‐dominated communities following fertilization. The specific relationship between SRA and traits‐abundance would be revised after the fertilizer was applied because the most limited resource for plants would change. For instance, soil nutrient limitation may be relieved when both N and P are added, the most limiting resource for plants is light in these fertilized communities (Li, Tian, Ren, Huang, & Zhang, 2013; Yang, Guo, Zhang, & Du, 2013. To test these hypothesis, we conducted a short‐term factorial experiment subjected to addition of nitrogen fertilizer (N), phosphorus fertilizer (P), or both of them (NP) in a Tibetan alpine meadow. We measured six traits including leaf economics spectrum (SLA, specific leaf area; LDMC, leaf dry matter content; LCC, leaf carbon concentration; LNC, leaf nitrogen concentration; LPC, leaf phosphorus concentration), the mature height (Hs) and the species above‐ground biomass for ten common species in each plot. This was because (1) the diversity of these individual traits indicate plant competition for different resources (Mason et al., 2012; Niu, He, Zhang, & Lechowicz, 2016; Niu, Messier, He, & Lechowicz, 2015), (2) abundance of common species reflect biotic and abiotic selection while occurrence of infrequent species is mostly controlled by various stochastic processes, for example, random drift and seed dispersal (Shipley, 2010; Wang, Wiegand, Kraft, Swenson, & Davies, 2016); (3) intraspecific variability resulted from the response of traits to fertilization even exceeds interspecific variability, especially for plant height and leaf nutrient content, which suggests that traits should be measured in each treatment or even each plot (Albert, Grassein, Schurr, Vieilledent, & Violle, 2011; Niu et al., 2014).

## 2 METHODS

### 2.1 Study site

The study was carried out at the Haibei National Field Research Station of Alpine Grassland Ecosystem (Haibei Station, 37°37′N, 101°12′E) in China, located in the northeastern part of the Tibetan Plateau at 3200 meters above sea level. The mean annual temperature is −1.7°C, ranging from −37.1°C to 27.6°C. The mean annual precipitation ranges from 426 to 860 mm, occurring mainly during the growing season from May to September. The annual mean air temperature and annual rainfall were 6.5°C and 461.4 mm during the growing season of 2011 and were 6.7°C and 339.9 mm during the growing season of 2012 (Wang, Liu, Chung, Yu, & Mi, 2014). The soil is classified as Mat–Gryic Cambisol (Chinese Soil Taxonomy Research Group, 1995). The alpine meadow community is dominated by *Kobresia humilis*,* Festuca ovina*,* Elymus nutans*,* Poa pratensis*,* Carex scabrirostris*,* Scirpus distigmaticus*,* Gentiana straminea*,* Gentiana lawrencei*,* Leontopodium nanum*,* Blysmus sinocompressus*,* Potentilla nivea*, and *Dasiphora fruticosa* (Luo et al., 2009).

### 2.2 Experimental design

A flat area was fenced by wire netting in May 2011 to prevent grazing by livestock in the enclosure. All visible animal wastes produced by grazing were cleaned up before the experiment was conducted in order to exclude the effect of animal wastes. Sixteen 3 m × 3 m plots (separated by 1 m) were randomly assigned to four treatments (four replicates each treatment). The four treatments were as follows: no fertilizer control, addition of N fertilizer (urea, 100 kg N·ha^−1^·year^−1^), addition of P fertilizer (granular triple super phosphate, 50 kg P·ha^−1^·year^−1^), and addition of NP (combination of N and P fertilizer, 100 kg N·ha^−1^·year^−1^ and 50 kg P·ha^−1^·year^−1^). Each plot was divided into four parts: for long‐term observation, for plant individual trait sampling, for plant abundance measurement, and for a possible additional treatment. The fertilizers were distributed by hand onto each plot. To make the fertilizers dissolve into the soil rapidly, we applied them in 15 July 2011 and in 22 June 2012 before the fine rain started.

### 2.3 Species trait and abundance measurements

Based on the experimental design from previous studies (Niu, He, & Lechowicz, 2016a; Niu et al., 2015), we chose ten common species in all plots to measure the key functional traits. These species accounted for 43%~76% of the above‐ground biomass in these communities. In each plot, we randomly measured the maximum height of five mature individuals (Hs) and sampled 20 mature undamaged leaves for each species to measure LDMC, SLA, and leaf nutrient. The ten leaves for each species in each treatment were scanned to measure leaf area, weighed to measure fresh mass and dry mass before and after dried at 65°C with a 10^−4^ g accuracy. For each leaf, SLA was calculated as the ratio of each leaf area to its dry mass, and LDMC was calculated as the ratio of dry mass to fresh mass. Finally, for each species in each plot, the dried leaves were ground up to measure leaf carbon concentration (LCC) and leaf nitrogen concentration (LNC) using an elemental analyser (2400 II CHN elemental analyser; Perkin‐Elmer, USA) and leaf phosphorus concentration (LPC) with a molybdate/stannous chloride method (He et al., 2009).

In late August 2012, that is, at the peak of plant biomass, standing biomass was harvested by species in a 0.5 × 0.5 m quadrat in each plot. The harvested biomass was oven‐dried at 65°C and then weighted with a 0.1 g accuracy.

### 2.4 Data analysis

In each quadrat, we calculated SRA as the biomass ratio of given species to the total above‐ground biomass of all species. For each species in each treatment, we averaged traits value and SRA, with 20 replicates (five individuals for each of four plots) for mature height and ten replicates for SLA and LDMC, but only four replicates for leaf nutrient and SRA from four plots.

A principal component analysis (PCA) was used to visualize the interrelationships among the five leaf economic traits and plant height for the common species in each treatment. Statistical significances of the correlations among traits were tested by Pearson correlation, and treatment effects were tested by ANOVA. Standard major axis (SMA) regressions were performed to test the relationship between SRA and individual trait (log‐transformed) in each treatment. A generalized linear model was also used to further evaluate the relationship between SRA and combinations of different traits in each treatment.

For each species, we estimated the response of SRA and individual trait to fertilization with a log response ratio [=log (SRA_fertilized_/SRA_control_)] and relative trait change [=(Trait_fertilized_ − Trait_control_)/Trait_control_)], respectively, where SRA_fertilized_ (or Trait_fertilized_) and SRA_control_ (or Trait_control_) are the mean SRA in plots with and without fertilizer addition, respectively. Thus, a positive value indicates that fertilization increases relative abundance (or trait) of a given species and vice versa (Li et al., 2013; Niu et al., 2008). Statistical significances of the response of SRA and traits were tested by pair *t‐*test on mean difference between fertilized and control treatment. All variables met the assumption of normality tested with Shapiro–Wilk tests and homogeneity of variances tested with Bartlett tests. All statistical analyses were performed in the software R (v 3.21, www.r-project.org).

## 3 RESULTS

### 3.1 Relationships among leaf economic traits and plant height

**Figure 1:**
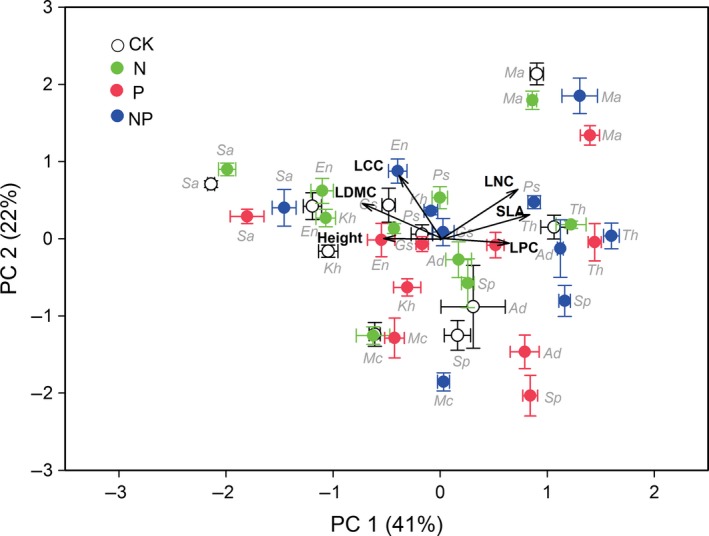
Principal component analysis correlation biplot (type‐II scaling) showing the relationships among five traits (black vectors) and plant height for common species in fertilized and control communities. SLA, specific leaf area; LDMC, leaf dry matter content; LCC, leaf carbon concentration; LNC, leaf nitrogen concentration; LPC, leaf phosphorous concentration. CK, unfertilized control; N, N fertilizer addition; P, P fertilizer addition; NP, addition of both N and P fertilizer. *Sa*,* Stipa aliena* Keng; *En*,* Elymus nutans* Griseb.; *Kh*,* Kobresia humilis* (C. A. Mey. ex Trautv.) Sergiev; *Ma*,* Medicago archiducis‐nicolai* Sirj.; *Th*,* Tibetia himalaica* (Baker) H. P. Tsui; *Gs*,* Gentiana straminea* Maxim; *Sp*,* Saussurea pulchra* Lipsch; *Mc*,* Morina chinensis* (Bat.) Diels; *Ad*,* Aster diplostephioides* (DC). C. B. Clarke., *Ps*,* Potentilla saundersiana* Royle

The first axis of PCA explained up 41% of the total variability in leaf economic traits and plant height while the second axis explained up 22% of that (Figure 1). The ordination diagram clearly reflected the divergence among species in leaf economic traits and plant height. As expected, mature height, LDMC, and LCC were negatively correlated with SLA and LPC (Figure 1), indicating species trade‐offs in resource conservation and competition versus resource acquisition for rapid growth. SLA was positively correlated with LNC (*r* = .75, *p *<* *.01, Figure 1) and LPC (*r* = .49, *p *<* *.01, Figure 1), while statured plant height was positively correlated with LDMC (*r* = .39, *p *<* *.01) and LCC (*r* = .51, *p *<* *.01).

Although individual trait significantly responded to fertilization (Fig. S1), the relationships among multiple traits did not significantly change with fertilization (*p *>* *.05, Figure 1). In response to fertilization, mature height increased, but LDMC and LCC decreased, for most species (e.g., *Aster diplostephioides*;* Potentilla saundersiana; Stipa aliena*; Fig. S1). N and P addition increased SLA, LNC, and LPC for most species (e.g., *Aster diplostephioides*;* Potentilla saundersiana; Elymus nutans*).

The responses of SRA to fertilization depended on species attributes (Fig. S2). Overall, relative abundance of grasses species (e.g., *Stipa aliena*;* Elymus nutans*) tended to increase, but forbs and legumes species (e.g., *Melilotoides archducis‐nicolai*;* Tibetia himalaica*) decreased following N addition (Fig. S1).

### 3.2 Relationships between species relative abundance and functional traits

**Figure 2:**
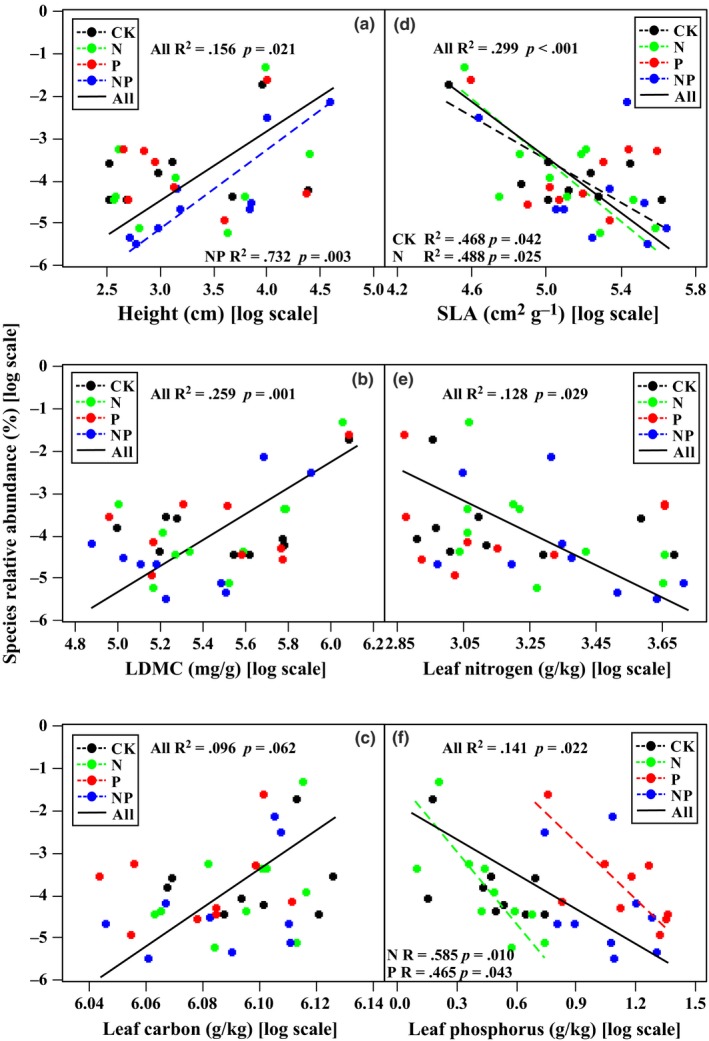
Relationships between species relative abundance and functional traits over common species in fertilized and unfertilized communities. *R* ^2^ and *p* values were estimated from standard major axis (SMA) regressions (detailed in Table S1). SLA, specific leaf area; LDMC, leaf dry matter content; CK, unfertilized control; N, N fertilizer addition; P, P fertilizer addition; NP, addition of both N and P fertilizer

Irrespective of fertilized treatments, SRA was positively correlated with plant height (*R* ^2^ = 0.16, *p *=* *.021, Figure 2a) and LDMC (*R* ^2^ = 0.26, *p *=* *.001, Figure 2b), but negatively correlated with SLA (*R* ^2^ = 0.30, *p *<* *.001, Figure 2d), LNC (*R* ^2^ = 0.13, *p *=* *.03, Figure 2e), and LPC (*R* ^2^ = 0.14, *p *=* *.02, Figure 2f).

When different treatments were separated, neither LDMC nor LCC or LNC were significantly correlated with SRA, but a stronger positive correlation between height and SRA was found in NP‐fertilized plots, while negative relationships between SRA and SLA or LPC were found in N or P fertilized plots (Table S1; Figure 2).

## 4 DISCUSSION

### 4.1 Species trade‐off in resource conservation and acquisition drives SRA

Our results showed that species were primarily differentiated along a trade‐off axis involving leaf economic traits and plant height. These findings were consistent with previous reports (He et al., 2009; Reich, 2014; Wright et al., 2004). As repeatedly documented in previous studies, larger SLA and higher leaf nutrient concentrations suggest higher rates of resource uptake and faster growth, while larger LDMC and longer lifespan indicate higher nutrient‐use efficiency and better competition for limited resources. Our results supported that a trade‐off between nutrient acquisitions versus conservation reflected by leaf traits existed among common species within local communities.

Taller species with larger LDMC and LCC, but lower SLA, LNC, and LPC, tends to be more abundant in fertilized communities. This result supported that species with higher resource conservation and competitive ability promote species abundance in competition‐dominated communities (Craine, Froehle, Tilman, Wedin, & Chapin, 2001; Sonnier, Navas, Fayolle, & Shipley, 2012). More importantly, this result also suggested that abundant species are often at the expense of resource acquisition and fast growth with low SLA and leaf nutrients (Arendonk & Poorter, 1994). Contrastingly, infrequent species may hold an advantage for dispersal to meta‐community or low competitive patch by seed and/or by clonal production (Liu et al., 2013; Niu, Schmid, Choler, & Du, 2012), because these species have higher capacity in uptaking limited resources and faster growth rate (indicating by high SLA and leaf nutrients). This partly explains why fast growth infrequent species diversity loss in fertilized communities but not in neighbor meta‐communities (Vellenda et al., 2013). Hence, it is possible that species occupy different positions along these trade‐offs in resource conservation versus acquisition and contribute to biodiversity maintenance in local communities or even to region scale.

### 4.2 The linkage between SRA and traits depends on competition for specific resource

Overall, our results show that the significance of trait‐abundance relationships depends on which fertilizer was added in community. This indicates that the importance of individual trait to SAR was tightly related with competition among species for most limited resource in communities. For instance, in both N and P fertilized communities, light availability becomes the most limited resource and competition for light drives SRA (Craine & Dybzinski, 2013), resulting a significant height‐abundance relationship. Similarly, when N fertilizer was added in plant communities, competition for soil available P and/or light resource become more important to community assembly, leading to significant correlations between SRA and SLA (and LPC). These correlations also supported hypothesis of grazing‐induced P‐limitation in this meadows (Niu et al., 2016a), which further explain why we find significant LPC‐abundance relation in these Tibetan alpine meadows: Abundance species in grazing rangeland are these with high leaf P content and fast growth rate (Niu, He, & Lechowicz, 2016b).

In short, significance of trait‐abundance relationships mainly depends on traits involved in competition for the most limited resource (Stanley Harpole & Tilman, 2006). Hence it is not surprising to find no significant trait‐abundance relationship when we combined several traits to predict SRA (Stanley Harpole & Tilman, 2006; Niu et al., 2012; Yang et al., 2013). Further work should pay more attentions on exploring the dependence of trait‐abundance relationship on environmental selection (Yan et al., 2013; Shipley et al., 2016) rather than assigning an unclear relation to neutral assembly (Clark, 2009).

### 4.3 Conclusion remark and limitation of our study

In short, our study found that species trade‐off in nutrient acquisition versus conservation is a key driver of SRA in Tibetan alpine meadow communities, while the linkage between SRA and traits depended on the most limited resources for plants (soil nutrient and/or light availability). The results support the importance of competitive exclusion among species in determining SRA especially in fertilized community. More importantly, to our best knowledge, we highlighted that common species have a higher competition resources ability at the expense of growth, while infrequent species hold an advantage in fast growth and contribute to biodiversity maintenance from local to meta‐community. However, balancing among complex operability in field experiment results in several limitations in our study. These are as follows: (1) although our hypothesis are applied to competition‐dominated communities, we selected fertilized communities rather than a controlled plant competition experiment and/or undisturbed “natural” community. This is mainly because previous studies have documented that competitive exclusion for light or soil nutrient drives community assembly occurred worldwide following nutrient additions (Rajaniemi, 2003); ii) as plant height and leaf traits (especially leaf nutrient) significantly responded to fertilization, we considered trait variations within species, and selected several common species in each treatment, which necessarily at the expense of sampling more infrequent species that are very rare in fertilized communities; (3) although our previous studies found that the nutrient content in roots is strongly correlated with that in leaves for common species in Tibetan alpine habitats (Geng, Wang, Jin, Liu, & He, 2014), it is still necessary to measure root traits for inferring plant competition for soil nutrient, which suggests that indications from leaf traits to plant competition for soil nutrient remain uncertain.

## CONFLICT OF INTEREST

None declared.

## AUTHOR CONTRIBUTIONS

J.‐S.H. and K.C.N. designed the study; H.Y.L. and Y.L. analyzed the data; F.R., L.L., and W.Y.Z. carried out the experiment; all co‐authors contributed to writing and discussions.
