# Supplementary material for: Trait‐abundance relation in response to nutrient addition in a Tibetan alpine meadow: The importance of species trade‐off in resource conservation and acquisition
Source: Ecol Evol. 2017 Nov 3;7(24):10575–81. doi: 10.1002/ece3.3439 (PMC5743641; doi:10.1002/ece3.3439)
Supplement: Supplementary file 1 [file ECE3-7-10575-s001.doc]

Supporting information

**Table S1.** Relationships between species relative abundance and functional traits estimated from Standard Major Axis regressions.

| SRA-Trait | Treatment | n | R^2^ | *P* | Slope | Intercept |
| --- | --- | --- | --- | --- | --- | --- |
| SRA and Hs | CK | 8 | 0.097 | 0.452 | 1.28 | -3.42 |
|  | N | 9 | 0.149 | 0.305 | 1.71 | -4.12 |
|  | P | 8 | 0.009 | 0.826 | 1.60 | -3.87 |
|  | NP | 9 | 0.732 | 0.003* | 1.85 | -4.62 |
|  | ALL | 34 | 0.156 | 0.021* | 1.65 | -9.36 |
| SRA and SLA | CK | 8 | 0.468 | 0.042* | -2.53 | 3.98 |
|  | N | 10 | 0.488 | 0.025* | -3.57 | 6.23 |
|  | P | 9 | 0.096 | 0 417 | -3.30 | 5.76 |
|  | NP | 9 | 0.185 | 0 .248 | -3.77 | 6.79 |
|  | ALL | 36 | 0.299 | <0.001* | -3.30 | 13.10 |
| SRA and LDMC | CK | 9 | 0.166 | 0.276 | 2.42 | -7.43 |
|  | N | 10 | 0.346 | 0.074 | 3.40 | -9.76 |
|  | P | 9 | 0.154 | 0.296 | 2.75 | -8.18 |
|  | NP | 9 | 0.308 | 0.121 | 3.55 | -10.07 |
|  | ALL | 37 | 0.259 | 0 .001* | 3.05 | -20.54 |
| SRA and LCC | CK | 9 | 0.075 | 0.475 | 36.47 | -98.17 |
|  | N | 10 | 0.138 | 0.291 | 57.86 | -154.80 |
|  | P | 9 | 0.065 | 0.507 | 42.54 | -113.94 |
|  | NP | 9 | 0.153 | 0.297 | 49.16 | -131.79 |
|  | ALL | 37 | 0.096 | 0.062 | 45.78 | -282.66 |
| SRA and LNC | CK | 9 | 0.096 | 0.418 | -3.00 | 2.49 |
|  | N | 10 | 0.304 | 0.098 | -4.77 | 5.08 |
|  | P | 9 | < 0.001 | 0.939 | 3.21 | -6.05 |
|  | NP | 9 | 0.265 | 0.156 | -4.75 | 5..05 |
|  | ALL | 37 | 0.128 | 0.029* | -3.82 | 8.47 |
| SRA and LPC | CK | 9 | 0.325 | 0.109 | -4.07 | -0.79 |
|  | N | 10 | 0.585 | 0.010* | -5.68 | -0.55 |
|  | P | 9 | 0.465 | 0.043* | -4.47 | 0.56 |
|  | NP | 9 | 0.134 | 0.333 | -5.87 | 0.82 |
|  | ALL | 37 | 0.141 | 0.022* | -2.74 | -1.80 |

**P* < 0.05.

Variable and trait codes: SRA, species relative abundance; Hs, saturated height; SLA, specific leaf area; LDMC, leaf dry matter content; LCC, leaf carbon concentration; LNC, leaf nitrogen concentration; LPC, leaf phosphorus concentration.

Supporting information to the paper

**Figure S1.** Species-specific functional traits responses to N addition, P addition and NP addition.

The trait responses are estimated by log response ratio of mean traits in fertilized plots relative to that in control plots. Hs: saturated height, SLA: specific leaf area, LDMC: leaf dry matter content, LCC: leaf carbon concentration, LNC: leaf nitrogen concentration, LPC: leaf phosphorus concentration. *Sa*: *Stipa aliena Keng*, *En*: *Elymus nutans Griseb*, *Kh*: *Kobresia humilis Sergievskaya*, *Ma*: *Melilotoides archducis-nicolai Yakovl*, *Th*: *Tibetia himalaica Tsui*, *Gs*: *Gentiana straminea Maxim*, *Sp*: *Saussurea pulchra Lipsch*, *Mc*: *Morina chinensis Diels*, *Ad*: *Aster diplostephioides Clarke*, *Ps*: *Potentilla saundersiana Royle*. Significance levels: ***: *P* < 0.001, **: *P* <0.01, *: *P*< 0.05. CK: unfertilized control, N: N fertilizer addition, P: P fertilizer addition, NP: addition of both N and P fertilizer.

**
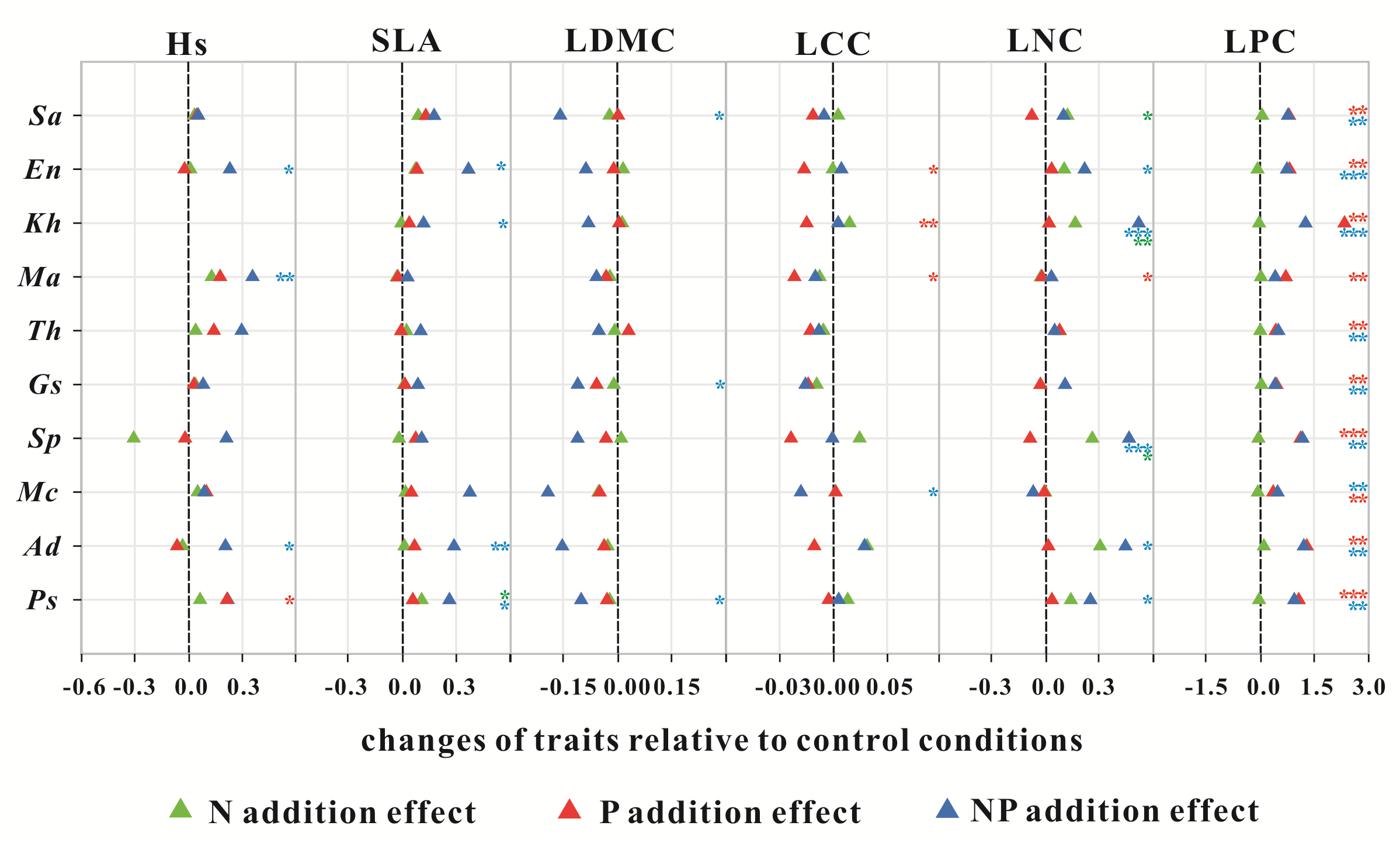
**

**Figure S2.** Species-specific relative abundance responses to N addition, P addition and NP addition. The a responses are estimated by log response ratio of mean species relative abundance in fertilized plots relative to that in control plots. Sa: Stipa aliena Keng, En: Elymus nutans Griseb, Kh: Kobresia humilis Sergievskaya, Ma: Melilotoides archducis-nicolai Yakovl, Th: Tibetia himalaica Tsui, Gs: Gentiana straminea Maxim, Sp: Saussurea pulchra Lipsch, Mc: Morina chinensis Diels, Ad: Aster diplostephioides Clarke, Ps: Potentilla saundersiana Royle. CK: unfertilized control, N: N fertilizer addition, P: P fertilizer addition, NP: addition of both N and P fertilizer.

**
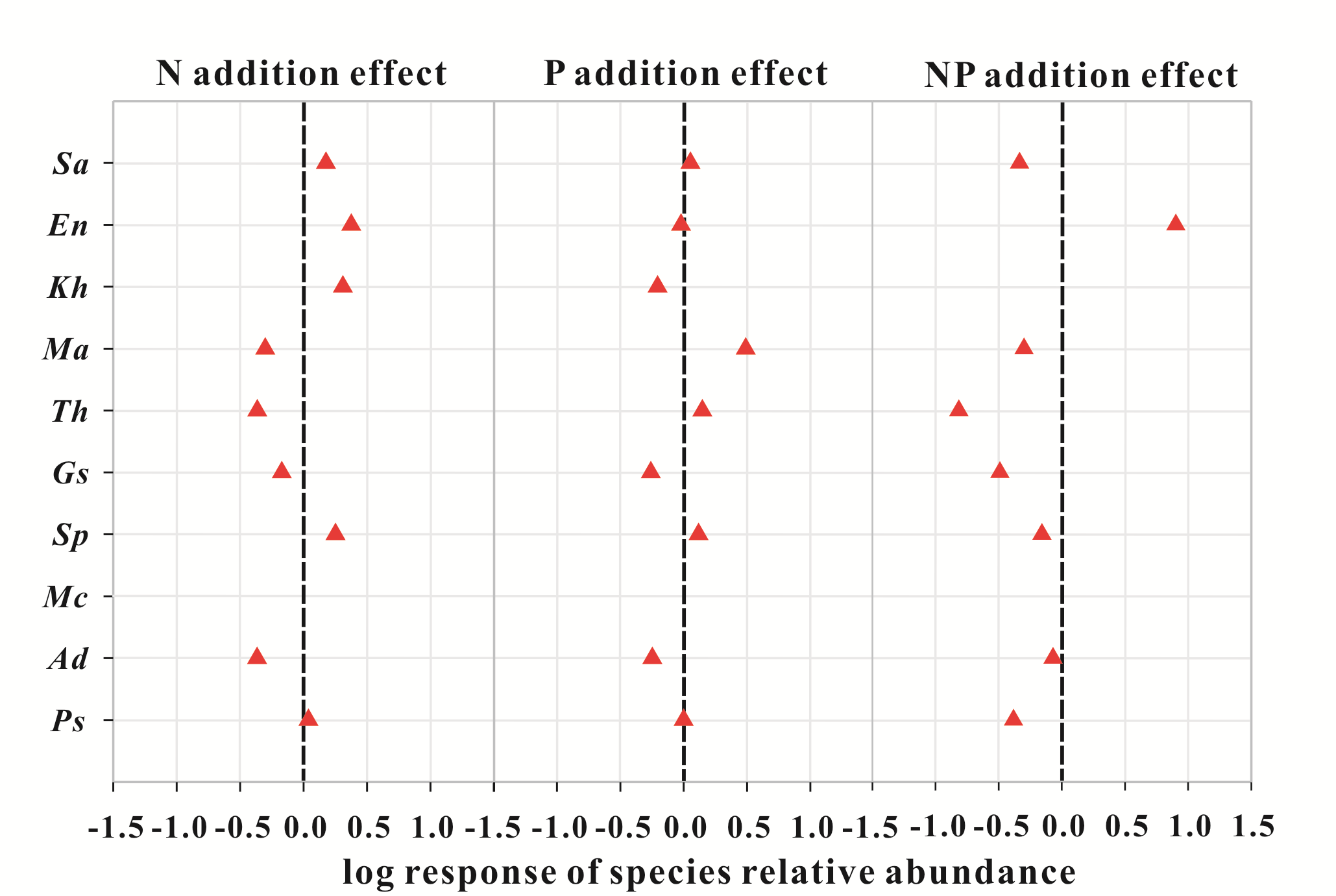
**
